# A Simulated Annealing Methodology to Multiproduct Capacitated Facility Location with Stochastic Demand

**DOI:** 10.1155/2015/826363

**Published:** 2015-03-05

**Authors:** Jin Qin, Hui Xiang, Yong Ye, Linglin Ni

**Affiliations:** ^1^School of Traffic and Transportation Engineering, Central South University, Changsha 410075, China; ^2^School of Traffic and Transportation Engineering, Changsha University of Science & Technology, Changsha 410076, China; ^3^Business Administration College, Zhejiang University of Finance & Economics, Hangzhou 310018, China

## Abstract

A stochastic multiproduct capacitated facility location problem involving a single supplier and multiple customers is investigated. Due to the stochastic demands, a reasonable amount of safety stock must be kept in the facilities to achieve suitable service levels, which results in increased inventory cost. Based on the assumption of normal distributed for all the stochastic demands, a nonlinear mixed-integer programming model is proposed, whose objective is to minimize the total cost, including transportation cost, inventory cost, operation cost, and setup cost. A combined simulated annealing (CSA) algorithm is presented to solve the model, in which the outer layer subalgorithm optimizes the facility location decision and the inner layer subalgorithm optimizes the demand allocation based on the determined facility location decision. The results obtained with this approach shown that the CSA is a robust and practical approach for solving a multiple product problem, which generates the suboptimal facility location decision and inventory policies. Meanwhile, we also found that the transportation cost and the demand deviation have the strongest influence on the optimal decision compared to the others.

## 1. Introduction

The facility location problem (FLP) is one of the most important models in the combinatorial optimization problem, which is to determine the number and locations of facilities and allocate customers' demands to these discrete located facilities in such a way that the total cost is minimized. Due to its wide application in numerous contexts such as regional planning, telecommunication and transportation infrastructure deployment, and energy management, a lot of problem variants and theirs models, which capture features of the real facility location problems, have been developed in the literature since the original formulation in Weber's work [1].

The FLP is NP-hard, which can be classified in two categories as capacitated problem and uncapacitated problem according to whether it includes the capacity of facilities. The capacitated facility location problem (CFLP) assumes that each facility can produce or ship limited quantities of the product and the demand of each customer is satisfied without violating the capacity restriction of any facility, which makes the CFLP more widely used, but also brings a lot of complexities to the problem.

Daskin [2] and Melo et al. [3] provided thorough reviews of the CFLP models. And we could found that many past models have several common characteristics, such as deterministic demands, a single product. Obviously, these models are insufficient to cope with many realistic capacitated facility location problems. So many extensions to the basic problems have been proposed and studied extensively.

Stochastic capacitated facility location problems have been proposed to approach situations in which some parameters are uncertain or stochastic. And in reality the demands are typically stochastic, which would bring risk to the inventory management. In order to deal with the stochastic demands and improve the service level, managers often hold some products as stock, which can result in increased inventory cost. In many contexts where product safekeeping is expensive, the inventory cost may account for a significant portion of the total system cost. So measuring the trade-off between the inventory cost and the service level is naturally very important in the facility location decision. However, inventory costs were not typically considered in many FLPs.

Inventory management and facility location are all major issues in the efficient design of a logistics network or supply chain (Gunasekaran et al., 2001 [4]; Pahl and Voß, 2014 [5]). However, literatures on supply chain optimization have traditionally considered these issues independently (e.g., [6–15]), which are not only because of different planning horizon but because of the computational complexity of the joint optimization problem.

Shen et al. [16] proposed a joint location model and restructured it into a set-covering model, and solutions only for two special cases were discussed; one is that variance of demand is proportional to the mean and the other is that demand has zero variance. Shu et al. (2005) [17] developed a more efficiency algorithm for the special cases. Shen [18] simplified the inventory cost of the products as the nonlinear function of the product quantity to the objective function and proposed the Lagrange algorithm to solve the problem; Shen and Qi (2007) [19] analyzed the transportation cost combined with the vehicle routing in the logistics network, but the order number was considered as a continuous variable in the formulation derivation. Kutanoglu and Lohiya [20] regarded the inventory cost as the linear function of product quantity. Ozsen et al. [21, 22] and Sourirajan et al. [23] proposed two different extensions to the model in Shen et al. [16]. Chen et al. [24] optimized facility locations, customer allocations, and inventory management decisions when facilities were subject to disruption risks. Hui et al. [25] studied the location-inventory results with joint replenishment (JR) and independent replenishment (IR), respectively, and found that the JR policy can obtain better solutions than IR policy.

In this paper, we study the stochastic multiproduct capacitated facility location problem (SMCFLP) that seeks the optimal solutions to minimize the total cost, including the facility setup cost, order cost, day-to-day shipment cost, and inventory cost, in which the demands for multiproducts are stochastic and satisfy the normal distribution. An open facility could serve one or more customers, while each customer could only be allocated to exactly one facility. The present work is motivated by a study of a local delivery service at a machinery manufacturing enterprise.

The remainder of the paper is organized as follows.  Section 2 presents some rational assumptions and notations of the SMCFLP. In Section 3, based on system cost analysis, we propose the model formulation that incorporates inventory control decision into multiproduct facility location problem with stochastic demand. In Section 4, an improved simulated annealing algorithm is developed to solve the model, and the setting of parameters is also discussed. In Section 5, the numerical example and the analysis of the results are given.

## 2. Notations and Assumptions

In formulating the optimization model, the following notations are used.

 
*i* denotes the candidate facility sites, *i* = 1,2,…, *I*.
 
*j* denotes the customer, *j* = 1,2,…, *J*.
 
*l* denotes the product, *l* = 1,2,…, *L*.
 
*λ*_*l*_ is the storage capacity occupation per unit product *l*.
 
*f*_*i*_ is the fixed setup cost of facility *i*.
 
*V*_*i*_ is the storage capacity of facility *i*.
 
*d*_*j*_^*l*^, *u*_*j*_^*l*^ are the mean and the standard deviation of the demand for product *l* of customer *j*, respectively.
 
*D*_*i*_^*l*^, *U*_*i*_^*l*^ are the mean and the standard deviation of the demand for product *l* of facility *i*, respectively.
 
*T*_*i*_^*l*^ is the lead time (in days); that is to say, the supplier takes the time *T*_*i*_^*l*^ to fulfill an incoming order from facility *i* for product *l*.
 
*Q*_*i*_^*l*^ is the fixed order quantity per order.
 
*h*_*i*_^*l*^ is the inventory cost per unit of product *l* of facility *i* (per day).
 
*o*_*i*_^*l*^ is the fixed cost of placing an order for product *l* in facility *i*.
 
*r*_*i*_^*l*^ is the transportation cost per unit to ship product *l* from the supplier to facility *i*.
 
*t*_*i*_^*l*^ is the elapsed time between two consecutive orders for product *l* in facility *i*.
 
*c*_*ij*_^*l*^ denotes the distribution cost per unit product *l* between the facility *i* and the customer *j*.
 
*δ*_1_, *δ*_2_ are the weight factors associated with transportation cost and inventory cost, respectively.
 
*α* is the united service level in the system, 0 < *α* < 1; namely, the fill rate of all demands for all products must not be less than *α* in all facilities.
 
*η* is the bank interest rate.
 
*μ* is discount rate (calculated by *r*).
 
*H* is the planning horizon (in year).
 
*M* is the maximum number of the facilities that are allowed to locate.

And we set two decision variables as

(1) Xi=1,if  a  faciliy  setup  on⁡  site  i;0,otherwise;Yijl=1,if  facility  i  serves  customer  j  for  product  l;0,otherwise.

Note that if *X*_*i*_ = 0, no products will pass facility *i* and vice versa.

According to the definition of the notations, we could know that

(2) $$\begin{array}{c} t_{i}^{l}=\frac{Q_{i}^{l}}{D_{i}^{l}}. \end{array}$$

And the fixed setup costs of the facilities *f*_*i*_, for all *i*, are all disposable, and the other costs are always invested per day, so in order to keep the consistency of all costs in time, the fixed setup costs should be shared in day in the planning horizon. And according to the above definitions, the discount rate *μ* could be calculated as follows:

(3) $$\begin{array}{c} \mu =\frac{1}{365}\overset{H}{\underset{i=1}{\sum }}\frac{\eta }{\left({\left(1+\eta \right)}^{i}-1\right)}. \end{array}$$

Some rational assumptions are proposed as follows.

1. Similar to Shen et al. [16], we assume that all stochastic customer demands *d*_*j*_^*l*^, for all *j*, *l*, are assumed to follow standard normal distribution and are independent of each other.
2. Each customer demand point is directly served by one and only one facility; namely, the demand of a customer could not be partitioned.
3. According to Shen et al. [16], we assume that all facilities have the same service level; in the other words, the fill rates for the demands in all facilities are identical in the system.
4. According to Ozsen et al. [22] and Chen et al. [24], we assume facility *i*, for all *i* using an economic order quantity (EOQ) model (*P*_*i*_^*l*^, *Q*_*i*_^*l*^) for inventory policy; that is to say, a fixed quantity *Q*_*i*_^*l*^ is ordered to the supplier, once the inventory quantity falls to or is below a reorder-point *P*_*i*_^*l*^.

According to assumption (1), for the rest of the paper, we could get the following evident relations:

(4) $$\begin{array}{c} D_{i}^{l}=\overset{J}{\underset{j=1}{\sum }}d_{j}^{l}Y_{ij}^{l},\;\forall i, \end{array}$$

(5) $$\begin{array}{c} U_{i}^{l}=\overset{J}{\underset{j=1}{\sum }}u_{j}^{l}Y_{ij}^{l},\;\forall i. \end{array}$$

From assumption (1), we also could know that the demands of all facilities are also stochastic and follow the standard normal distribution.

## 3. Model Formulation

According to assumption (4), the facility *i* performs (*P*_*i*_^*l*^, *Q*_*i*_^*l*^) inventory policy to meet the stochastic demand pattern. But even if the order is triggered, the ordered products could be received after *T*_*i*_^*l*^ days. So once an order is submitted, the inventory products should cover the demand produced during lead time *T*_*i*_^*l*^ with a given probability *α*. This probability *α* is known as the level of service for the system or the fill rate for the demand. So the service level constraints in the facility *i* can be expressed as follows:

(6) $$\begin{array}{c} P\left(D\left(T_{i}^{l}\right)\leq P_{i}^{l}\right)=\alpha ,\;\forall i,l, \end{array}$$

where *D*(*T*_*i*_^*l*^) is the stochastic demand for product *l* in facility *i* produced with the lead time *T*_*i*_^*l*^.

According to Shen et al. [16, 18], we could know the average inventory cost rate of product *l* in facility *i* as

(7) $$\begin{array}{c} h_{i}^{l}Z_{\alpha }\sqrt{T_{i}^{l}}\sqrt{U_{i}^{l}}+\frac{h_{i}^{l}Q_{i}^{l}}{2}, \end{array}$$

where *Z*_*α*_ is the value of standard normal variate, which accumulates a probability of *α*.

The operation cost for product *l* in facility *i* during the period is

(8) $$\begin{array}{c} r_{i}^{l}Q_{i}^{l}+o_{i}^{l}+\left(h_{i}^{l}Z_{\alpha }\sqrt{T_{i}^{l}}\sqrt{U_{i}^{l}}+\frac{h_{i}^{l}Q_{i}^{l}}{2}\right)t_{i}^{l}. \end{array}$$

We could get the operation cost rate of product *l* in facility *i*, based on expression 9 divided by *t*_*i*_^*l*^ (*t*_*i*_^*l*^ = *Q*_*i*_^*l*^/*D*_*i*_^*l*^):

(9) $$\begin{array}{c} \left(r_{i}^{l}+\frac{o_{i}^{l}}{Q_{i}^{l}}\right)D_{i}^{l}+h_{i}^{l}Z_{\alpha }\sqrt{T_{i}^{l}}\sqrt{U_{i}^{l}}+\frac{h_{i}^{l}Q_{i}^{l}}{2}. \end{array}$$

So the total operation cost rate is

(10) $$\begin{array}{c} \overset{I}{\underset{i=1}{\sum }}\overset{L}{\underset{l=1}{\sum }}\left(h_{i}^{l}Z_{\alpha }\sqrt{T_{i}^{l}}\sqrt{U_{i}^{l}}+\frac{h_{i}^{l}Q_{i}^{l}}{2}\right)+\overset{I}{\underset{i=1}{\sum }}\overset{L}{\underset{l=1}{\sum }}\left(r_{i}^{l}+\frac{o_{i}^{l}}{Q_{i}^{l}}\right)D_{i}^{l}. \end{array}$$

The transportation cost rate of the entire system is

(11) $$\begin{array}{c} \overset{I}{\underset{i=1}{\sum }}\overset{J}{\underset{j=1}{\sum }}\overset{L}{\underset{l=1}{\sum }}c_{ij}^{l}d_{j}^{l}Y_{ij}^{l}. \end{array}$$

The total fixed setup cost of the system is

(12) $$\begin{array}{c} \overset{I}{\underset{i=1}{\sum }}f_{i}X_{i}. \end{array}$$

As mentioned before, the fixed setup cost is one-time expenditure in the planning horizon, but the other costs are counted by day, so the setup cost should be converted into day-cost in order to be consistent with all costs in unit:

(13) $$\begin{array}{c} \mu \overset{I}{\underset{i=1}{\sum }}f_{i}X_{i}. \end{array}$$

Therefore, the total cost rate of the system is

(14) μ∑i=1IfiXi+∑i=1I∑l=1Lδ1hilZαTilUil+hilQil2  +∑i=1I∑j=1Mδ2cijl+ril+oilQilDil.

The objective of the problem is to minimize the cost 15. But there are too many uncertain variables in it, which could make the solution approach difficult to be designed.

Assume that the cost function 15 is continuously differentiable on order quantity, then performing differentiation on it in terms of *Q*_*i*_^*l*^, for all *i*, *l*, and letting the derivation be equal to zero (minimizing the total cost in a centralized approach), we could obtain

(15) $$\begin{array}{c} \delta_{1}\frac{h_{i}^{l}}{2}-\delta_{2}\frac{o_{i}^{l}}{{\left(Q_{i}^{l}\right)}^{2}}D_{i}^{l}=0,\;\forall i,l. \end{array}$$

Then we could get the optimal order quantity of product *l* per order in facility *i* as follows:

(16) $$\begin{array}{c} {\overset{-}{Q}}_{i}^{l}=\sqrt{\frac{2\delta_{2}o_{i}^{l}D_{i}^{l}}{\delta_{1}h_{i}^{l}}},\;\forall i,l. \end{array}$$

Replacing 4, 5, and 16 in expression 14, the cost function 15 can be expressed as follows:

(17) μ∑i=1IfiXi+∑i=1I∑l=1Lδ1hilZαTil∑j=1JujlYijl  +∑i=1I∑l=1L2δ1δ2hiloil∑j=1JdjlYijl  +∑i=1I∑j=1J∑l=1Lδ2cijl+rildjlYijl.

So, we could present the model to solve the SMCFLP considering the inventory cost as follows:

(18) min⁡⁡Φ=μ∑i=1IfiXi +∑i=1I∑l=1Lδ1hilZαTil∑j=1JujlYijl +∑i=1I∑j=1J∑l=1Lδ2cijl+rildjlYijl +∑i=1I∑l=1L2δ1δ2hiloil∑j=1JdjlYijl

subject to

(19) $$\begin{array}{c} \overset{I}{\underset{i=1}{\sum }}Y_{ij}^{l}=1,\;\forall j,l, \end{array}$$

(20) $$\begin{array}{c} \overset{J}{\underset{j=1}{\sum }}\overset{L}{\underset{l=1}{\sum }}\lambda_{l}d_{j}^{l}Y_{ij}^{l}\leq V_{i}X_{i},\;\forall i, \end{array}$$

(21) $$\begin{array}{c} Y_{ij}^{l}\leq X_{i},\;\forall i,j,l, \end{array}$$

(22) $$\begin{array}{c} \overset{I}{\underset{i=1}{\sum }}X_{i}\leq M, \end{array}$$

(23) $$\begin{array}{c} X_{i}\in \left\{0,1\right\},\;\forall i,j,l, \end{array}$$

(24) $$\begin{array}{c} Y_{ij}^{l}\in \left\{0,1\right\},\;\forall i,j,l. \end{array}$$

The model would determine where to open facilities no more than *M* in *I* candidate sites and how to serve *J* customers with stochastic demand under service level constraint. The objective function 18 is to minimize the total system cost, including location cost, inventory cost, order cost, and transportation cost. Constraint 19 states that each demand must be only allocated to one facility. Constraint 20 ensures that the total demands allocated to a certain facility should not exceed its capacity. Constraint 21 restricts a demand only to be allocated to an open facility. Constraint 22 is the maximum restriction of the opened facilities. Constraints 23 and 24 are the standard integrality constraints, and *Y*_*ij*_^*l*^ ∈ {0,1} representing single-sourcing constraints, which means that all of the demands of a customer must be assigned to the same facility.

## 4. Solution Approach

The above SMCFLP model 19–24 is a large-scale nonlinear mixed-integer programming model, which is NP-hard problem [16]. We were able to easily compute optimal solutions for small problem instances. However, as the number of products, number of facilities, and number of customers approach some practical size, the attractiveness of this methodology to provide optimal solutions to such practical sized problems in a reasonable amount of time deteriorates considerably. Its NP-hard nature makes the exact algorithms only for small problems and heuristics the natural choice for larger instances. There was a long list of works on designing heuristics algorithms for this problem over the years.

The Lagrangian method (Miranda and Garrido, 2008 [26]; Nezhad et al, 2013 [27]) and branch and bound algorithm [28, 29] are always used to solve the similar problem. But the Lagrangian method and branch and bound method are intuitive, inflexible, and complicated. In order to increase the adaptability of the solution method, we therefore began testing heuristic approaches to this combinatorial problem. Several approaches were tried including genetic algorithm (GA) and simulated annealing (SA) algorithm.

Kirkpatrick et al. [30] introduced the SA; then, it has proved to be an effective tool for approximating globally optimal solutions to many NP-hard optimization problems. We adopted the SA procedure because of its ability to quickly combine the facility location decision and inventory control decision into a single large problem. In the last 30 years, SA has been applied to many optimization problems in a wide variety of areas such as facility layout [31–33], job scheduling [34–36], and network design ([37–43]).

The solution of SMCFLP includes two parts, *X*_*i*_ and *Y*_*ij*_, where *X*_*i*_ denotes whether or not to open the facility in site *i* and *Y*_*ij*_ denotes the service allocation of customers' demand. The two variables are interdependent and interactional. As each demand must be allocated to an opening facility, we could think that *Y*_*ij*_ is determined by *X*_*i*_. This relation also can be seen from constraint 22 in the model.

According to the above characteristics of the problem, we could use the combined simulated annealing (CSA; [43]) algorithm to solve the model. The CSA works in two layers as the outer layer algorithm (OLA) and the inner layer algorithm (ILA), in which the OLA optimizes the facility location decision. Then, the ILA optimizes the demand allocation decision based on the determined facility location decision determined in the OLA. In each layer the SA is used and the combination of them is CSA.

It is well known that the neighboring function is crucial to the good performance of the SA. In the algorithm we should design different neighboring functions in OLA and ILA, respectively.

The OLA would optimize the facility location decision. So the neighboring function of the OLA to modify the configuration of the current solution and generate a neighboring solution could have three different operations.

1. If the number of the opened facility is less than the allowed maximum *M*, then select a candidate site *i* which satisfies *X*_*i*_ = 0 in current solution *S* randomly and set *X*_*i*_ = 1; namely, a facility is located in the site *X*_*i*_.
2. If the number of the opened facility is no less than 1, then select a site *i* which satisfies *X*_*i*_ = 1 in current solution *S* randomly and set *X*_*i*_ = 0; namely, the opened facility as site *X*_*i*_ is closed.
3. Exchange two facilities with different status; namely, select a site *i* which satisfies *X*_*i*_ = 0 and another site *i*′ which satisfies *X*_*i*′_ = 1 in current solution *S*, and then set *X*_*i*_ = 1 and *X*_*i*′_ = 0.

In the implementation of the OLA, we could only select one operation from the above three operations to perform the neighboring function each time. And after implementing this OLA operation, it should allocate the customers' demand to the opened facilities again.

ILA is to determine the demand allocation decision. According to the features of the allocation decision, there are two operations to generate the neighboring solutions in the ILA.

1. Select two allocation variables *Y*_*ij*_^*l*^ and *Y*_*ij*′_^*l*^ that satisfy *Y*_*ij*_^*l*^ = 1 and *Y*_*ij*′_^*l*^ = 0; then set *y*_*ij*′_ = 1, *y*_*ij*_ = 0; namely, it allocates the demand of customer *i* for product *l* from facility *j* to another facility *j*′.
2. Exchange the facilities which service two customers, respectively, with each other. To be specific, select four allocation variables as *y*_*i*_1_*j*_1__ = 1, *y*_*i*_2_*j*_2__ = 1, *y*_*i*_1_*j*_2__ = 0, and *y*_*i*_2_*j*_1__ = 0; then set *y*_*i*_1_*j*_2__ = 1, *y*_*i*_2_*j*_1__ = 1, *y*_*i*_1_*j*_1__ = 0, and *y*_*i*_2_*j*_2__ = 0.

Similarly, the neighboring function of the ILA could select only one operation to perform each time. In addition, it should ensure that demands of all customers must be satisfied and the facilities have no capacity violations that exist in the neighboring solution. Otherwise, it should return to the old solution and reselect an operation to perform.

In addition, it is well known that the SA algorithm must start with an initial solution or with a solution produced using a heuristic. In this work, we use the randomly generated initial solution, which is similar to that in Qin et al. [43].

Let Φ(*S*) denote the objective function value (total cost) of solution *S*; then, the steps of the CSA could be given as follows.

*Step 0 (initialization).* Set the initial temperature *t*, the cooling rate *ξ*, the stop temperature *t*_*f*_, and the maximum iteration number *N* at each temperature value. Set counter number *K*_1_ = 1, *K*_2_ = 1.

*Step 1 (generate initial solution).* The initial solution *S* could be generated by randomly allocating customers' demands to the facilities. Let the global optimal solution $\overset{-}{S}=S$.

*Step 2 (check feasibility).* The method now checks the demand allocations to ensure no capacity violations exist. The demands of the customers are also checked. If the solution *S* is not feasible, we should return to Step 1.

*Step 3 (generate a feasible neighboring solution).* Perform the outer layer neighboring function on *S*, and get the new feasible solution *S*′.

*Step 4 (perform ILA)*

*Step 4.1.* Regard solution *S*′ as the initial solution and the current optimal solution in the ILA; then, generate the ILA neighboring solution *S*′′ based on solution *S*′.

*Step 4.2.* If Φ(*S*′′) < Φ(*S*′), then set *S*′ = *S*′′; otherwise, generate a random number *ρ*′ from (0,1), if *ρ*′ < exp⁡(−(Φ(*S*′′) − Φ(*S*′))/*t*); then, set *S*′ = *S*′′.

*Step 4.3.* Increase the counter number as *K*_2_ ← *K*_2_ + 1.

*Step 4.4.* If *K*_2_ ≤ *N*, then go to Step 4.1; otherwise, set *K*_2_ = 1, stop the ILA computation, and return to the OLA.

*Step 5 (update the global optimal solution).* If $\Phi (S^{\prime })<\Phi (\overset{-}{S})$, then set $\overset{-}{S}=S^{\prime }$.

*Step 6 (evaluate current solution and examine metropolis condition).* If Φ(*S*′) < Φ(*S*), then set *S* = *S*′, or else generate a random number *ρ* ∈ (0,1), if *ρ* < exp⁡[−(Φ(*S*′) − Φ(*S*))/(*t*)], set *S* = *S*′.

*Step 7 (increment counters).* One has *K*_1_ ← *K*_1_ + 1. If *K*_1_ ≤ *N*, then return to Step 3; otherwise, proceed to Step 8.

*Step 8 (adjust temperature).* Adjust temperature by the cooling rate *ξ*. Mathematically this is *t* ← *t* · *ξ*. And set *K*_1_ = 1; then return to Step 3.

*Step 9 (convergence check).* If the temperature *t* is greater than or equal to the given stopping value *t*_*f*_, then go to Step 3; otherwise, stop the computation and output the optimal solution $\overset{-}{S}$.

Note that Step 5 is to save the global optimal solution that has been searched so far in the algorithm. This operation does not take the acceptance probability into consideration. So the algorithm could avoid the missing of the global optimal solution possibly.

## 5. Numerical Examples

To evaluate the performance of CSA algorithm on the problem, several problems with different sizes were developed. We varied the values of several parameters of problem and heuristic. The problem parameters are described by the number of candidate facilities *I*, the number of customers *J*, and the number of products *L*. The heuristic parameter is described by the coiling rate *ξ*.

Here we use 20 instances with different sizes as benchmark problems. The stopping temperature value *t*_*f*_ = 0.0001. The planning horizon time *H* = 10 years, the bank interest rate *η* = 0.04, and the service level *α* in all facilities is equal to 0.95. The weight factor of the inventory cost and transportation cost is *δ*_1_ = 2 and *δ*_2_ = 1, respectively. The other data are drawn from the certain range randomly; for example, the capacity values of the facilities are chosen randomly from the range [10000, 20000].

The model and CSA were implemented in Visual C# 2008. Computational experiments were performed on the personal computer with CORE I5-3317U (1.7 Ghz) CPU and 4 G RAM.

The setting of different parameters and computational results of these instances are reported in Table 1. Each instance was computed 10 times and the optimal solution is chosen as the results (the OFV is the objective function value Φ).

It can be seen from Table 1 that the CSA could find the near optimal solutions for all instances with different sizes. It is clear that the bigger value of cooling rate could provide more benefit to the larger size problem.

The quality of the optimal solution found by the local search algorithm may be dependent on its initial solution. In order to test the model and the algorithm, we solved ten optimal solutions with different initial solutions for the largest instance, in which the system includes 18 candidate facility sites, 60 customers, and 5 products.  Table 2 shows the data.

Computational results based on the ten different initial solutions are reported in Table 2. We can find that even the initial solutions are different, the objective functions values Φ of the optimal solutions obtained by the CSA algorithm are very close, and the gap between the maximal value (7592152.387) and the minimum value (7390387.970) is only 2.73%. Compared with the initial solutions, the optimal objective values have 15.28%~34.94% cost saving. So it can prove the proposed optimal model and algorithm is rational and available.

**Figure 1:**
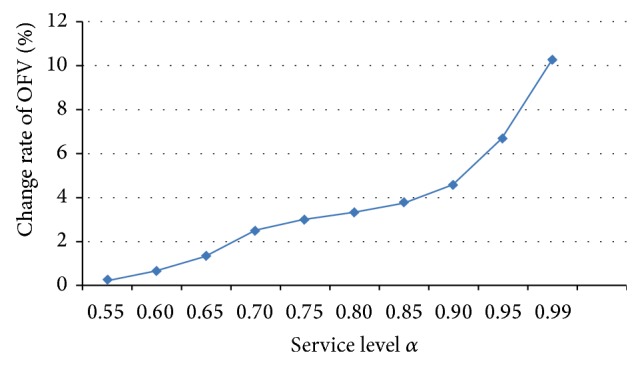
OFV versus service level *α*.

Figure 1 shows the change rate of OFVs when the service level *α* varied from 0.5 to 0.99. It is obvious that the OFVs increase when the service level rises. Also the greater the service level is, the faster the OFV increases. This case accords with the fact perfectly.

**Figure 2:**
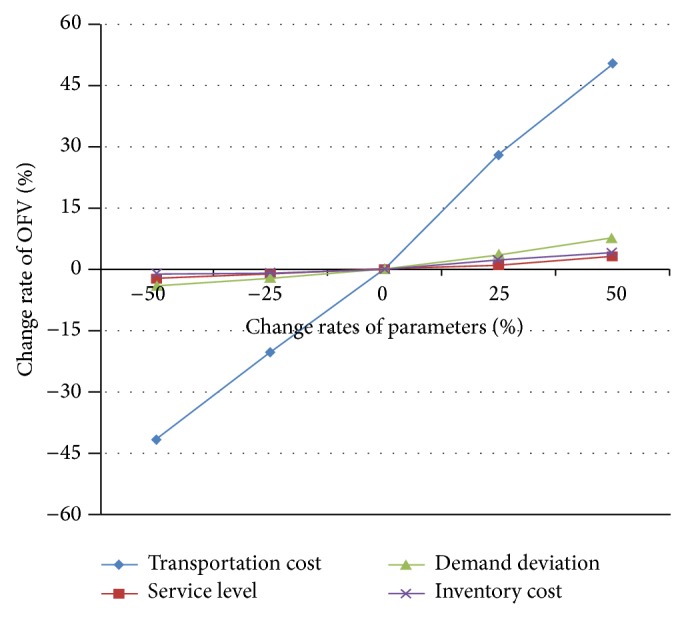
OFV versus parameters.

Figure 2 illustrates that the relative change rate of OFV, compared with all the unit transportation costs, the unit inventory costs, and the demand deviations, changes with the same magnitude, respectively. We could find that the transportation cost has the most impact on the system, and when its magnitude of change varied from –50% to 50%, the change rate of the OFV exceeds 90%, which is beyond the impaction of inventory cost and service level on the OFV by far. Meanwhile, the change rate of the OFV is about 12% when the demand deviation varied.

It should be noted that the result is found under the condition of the weight factor of the inventory cost *δ*_1_ twice as large as the weight factor of the transportation cost *δ*_2_. So we could consider the transportation cost is the most important factor in the logistics system cost.

From the above analysis, we could find that the service level, the demand deviation, the inventory cost, and the transportation cost all could have influence on the total system cost, which are directly proportional to the cost. But the demand deviation and the transportation cost have more influence on the system cost in contrast with other factors. So from the managers' point of view, if they want to save their logistics system cost, they should pay more attention to selecting the most economic transportation mode and predict the demand more accurately.

## 6. Conclusions

In this paper, we analyzed the stochastic multiproduct capacitated facility location problem with stochastic demand and service-level constraint. A nonlinear mixed-integer programming model is proposed, in which the objective is to minimize the total system cost, including fixed facility setup cost, inventory cost, order cost, and transportation cost, under the precondition of satisfying the certain service level.

The CSA solution method is developed to solve the large-scale problem, which is divided into two layers: the outer subalgorithm and the inner subalgorithm. The outer subalgorithm optimizes the facility location decision, and the inner subalgorithm optimizes the demand allocation based on the determined facility location decision. This method divides the problem into two layers to be solved; that is to say, the solution space in each iteration random search of the algorithm is also divided into smaller parts; thus the probability of obtaining the optimal solution of the problem in each iterative computation is raised.

We offer two important contributions to the SA application. First, we extend the breadth of applications by studying a new facility location problem, involving multiproduct and stochastic demand. Second, we developed a bilevel SA algorithm to solve the problem, in which computational performance was systematically evaluated under a variety of problem scenarios and control parameter settings.

Computational results show the CSA is a robust and practical approach for solving a multiple product problem, which could provide scientific guidance for the stochastic multiproduct facility location problem. Furthermore, the influences of the parameters (service level, demand deviation, unit inventory cost, and unit transportation cost) on the total system cost are investigated. We found that transportation cost and the demand deviation could have more influence than the other factors on the total cost (or the optimal decision).

## Figures and Tables

**Table 1:** Computational results of different sizes.

Number	Size	Cooling rate	Performance	
	*I* × *J* × *L *	*ξ*	OFV	CPU time (s)
1-1	9 × 30 × 3	0.20	3105834.534	12.010
1-2	9 × 30 × 3	0.40	3041586.681	12.957
1-3	9 × 30 × 3	0.60	3026234.953	13.647
1-4	9 × 30 × 3	0.80	**3025685.852**	15.211
1-5	9 × 30 × 3	0.95	3025736.583	13.254
2-1	9 × 60 × 5	0.20	6212414.241	16.124
2-2	9 × 60 × 5	0.40	6125510.853	21.631
2-3	9 × 60 × 5	0.60	6123912.581	20.684
2-4	9 × 60 × 5	0.80	6112683.749	24.255
2-5	9 × 60 × 5	0.95	**6103823.625**	27.218
3-1	18 × 30 × 3	0.20	4362573.568	21.115
3-2	18 × 30 × 3	0.40	4013215.824	23.644
3-3	18 × 30 × 3	0.60	3983564.525	28.592
3-4	18 × 30 × 3	0.80	3963247.423	29.297
3-5	18 × 30 × 3	0.95	**3962173.914**	30.582
4-1	18 × 60 × 5	0.20	7556954.122	39.369
4-2	18 × 60 × 5	0.40	7438845.631	43.251
4-3	18 × 60 × 5	0.60	7429689.169	44.258
4-4	18 × 60 × 5	0.80	7421958.561	47.914
4-5	18 × 60 × 5	0.95	**7390387.970**	52.899

**Table 2:** Objective function value of initial and optimal solutions.

Number	OFVs of initial solutions	OFVs of optimal solutions	Gap^1^ (%)	Gap^2^ (%)
1	9626812.58	7514417.038	21.94	1.68
2	10390043.06	7454652.213	28.25	0.87
3	8848002.052	7496323.639	15.28	1.43
4	9669135.964	7444631.243	22.90	0.73
5	9559931.798	7570938.775	20.81	2.44
6	10681795.74	7461077.196	30.15	0.96
7	9235880.133	7454163.686	19.29	0.86
8	9928204.532	**7390387.970**	25.56	0
9	11669115.5	***7592152.387***	34.94	2.73
10	8848396.408	7418253.878	16.16	0.38

Gap^1^: gaps between OFVs of initial solutions and optimal solutions.

Gap^2^: gaps between OFVs of optimal solutions and the minimum value of optimal solutions.
